# A sequence-specific, nanoparticle-based biosensor platform for rapid and visual identification of serum hepatitis B virus pregenomic RNA in chronic hepatitis B patients

**DOI:** 10.1128/spectrum.01918-25

**Published:** 2025-12-18

**Authors:** Yuanyuan Gu, Qi Zhao, Yan Tan, Junfei Huang, Yi Wang, Shijun Li, Xu Chen

**Affiliations:** 1 The Second Clinical Medical College, Guizhou University of Traditional Chinese Medicine 326770 , Guiyang, Guizhou, People’s Republic of China; 2 Medical Science Laboratory of Integrative Chinese and Western Medicine, The Second Affiliated Hospital, Guizhou University of Traditional Chinese Medicine 643558, Guiyang, Guizhou, People’s Republic of China; 3 Clinical Laboratory, Guizhou Provincial Center for Clinical Laboratory, Guiyang, Guizhou, People’s Republic of China; 4 Experimental Center, Guizhou Provincial Center for Disease Control and Prevention, Guiyang, Guizhou, People’s Republic of China; 5 Experimental Research Center, Capital Center for Children’s Health, Capital Medical University, Capital Institute of Pediatrics 36776, Beijing, People’s Republic of China; University of Pretoria, Pretoria, Gauteng, South Africa

**Keywords:** chronic hepatitis B, pregenomic RNA, loop-mediated isothermal amplification, restriction endonuclease, real-time fluorescence detection, biosensor

## Abstract

Chronic hepatitis B virus (HBV) infection is a major cause of liver-related morbidity and mortality worldwide. Serum HBV pregenomic RNA (pgRNA) is a surrogate marker for the transcriptional activity of covalently closed circular DNA. Here, we successfully designed a novel point-of-care (POC) diagnostic platform that allows specific, sensitive, rapid, and visual identification of HBV pgRNA. The platform integrates probe-based reverse transcription loop-mediated isothermal amplification (RT-LAMP) with either **r**estriction **e**ndonuclease-mediated real-time **f**luorescence (REF) or a gold nanoparticle-based lateral flow biosensor (AuNPs-LFB), termed HBV-RT-LAMP. A unique set of probe-based LAMP primers targeting HBV-pgRNA was successfully designed. The optimal conditions for HBV-RT-LAMP were determined to be 64°C and 30 min. AuNPs-LFB and a pocket fluorescence detector (REF assay) were used for readout of the products. Our assay detected the target gene at concentrations as low as 50 copies/mL of HBV RNA standard and did not produce cross-reactions with HBV DNA (treated with DNase I) or other pathogens. The entire detection process, including HBV RNA extraction (45 min), LAMP (30 min), and the interpretation of results (AuNPs-LFB, less than 2 min), could be performed within 80 min, with no need for expensive devices. Therefore, the HBV-RT-LAMP diagnostic system developed in this study can potentially serve as a useful POC diagnostic tool for the evaluation of chronic HBV infection status and antiviral drug efficacy.

**IMPORTANCE:**

Chronic hepatitis B (CHB) is still a serious global concern that can result in severe liver-related diseases, including liver cirrhosis and hepatocellular carcinoma. Serum hepatitis B virus (HBV)-pregenomic RNA (pgRNA) has been proposed as a surrogate intrahepatic covalently closed circular DNA marker in CHB patients. Here, for the first time, a novel point-of-care diagnostic platform, termed HBV-reverse transcription loop-mediated isothermal amplification (RT-LAMP), which integrates probe-based RT-LAMP with either restriction endonuclease-mediated real-time fluorescence (REF) detection or a gold nanoparticle-based lateral flow biosensor (AuNPs-LFB), was developed and applied for accurate, sensitive, specific, rapid, and visual identification of HBV-pgRNA.

## INTRODUCTION

Chronic hepatitis B virus (HBV) infection is a significant global health problem affecting more than 296 million patients worldwide and resulting in nearly 820,000 annual deaths due to the development of progressive liver-related diseases such as cirrhosis and hepatocellular carcinoma (HCC) (1). Covalently closed circular DNA (cccDNA) serves as the HBV viral reservoir in hepatocytes and represents a primary component of the HBV lifecycle (2, 3). Therefore, the identification of the presence and activity of HBV cccDNA in liver tissue can inform decisions regarding chronic hepatitis B (CHB) treatment options. However, direct detection of HBV cccDNA in liver tissue presents significant clinical challenges, primarily due to the invasive nature of liver biopsy procedures, which carry risks of potential complications (4, 5). Additional limitations include the subjectivity of different technicians and low specimen yield (6). Recent studies have confirmed that serum HBV RNA in patients with CHB is primarily composed of non- or partially reverse-transcribed pregenomic RNA (pgRNA) and rendered in HBV virion-like particles (7, 8). HBV pgRNA is derived from the active transcription of cccDNA in infected hepatocytes (9, 10). More importantly, the serum HBV pgRNA in CHB patients receiving nucleos(t)ide analog (NA) treatment can still accurately reflect cccDNA activity when reverse transcription and synthesis of HBV DNA are inhibited (9, 11). Hence, HBV pgRNA in serum is recognized as a good surrogate biomarker for HBV cccDNA activity in infected hepatocytes.

Current diagnostic approaches targeting serum HBV pgRNA predominantly utilize polymerase chain reaction (PCR)-based methodologies, which are indispensable in hepatitis management and therapeutic interventions due to their high specificity and sensitivity (12–14). Despite their efficacy, these approaches are often unaffordable and inaccessible in resource-constrained regions because they require expensive thermocycling instruments and trained technical personnel. The World Health Organization (WHO) estimated that low-income countries (LICs) face a high prevalence of HBV infection due to limited availability of detection, prevention, and management (15, 16). The incidence and prevalence of HBV infection are 9.2- and 7.4-fold higher, respectively, in LICs than in high-income countries (HICs) (16). In addition, the HBV diagnosis rate plummets from 18% in HICs to merely 0.8% in LICs (16). Hence, developing a user-friendly, simple, affordable, sensitive, specific, and rapid point-of-care (POC) diagnostic platform for accurate screening of CHB is critical for achieving the WHO’s goal of eliminating HBV infection by 2030.

Loop-mediated isothermal amplification (LAMP) is a transformative nucleic acid diagnostic technology known for its high efficiency and isothermal properties (17, 18), which employs a set of 4–6 primers targeting 6–8 unique segments of the desired sequence and *Bacillus stearothermophilus* (*Bst*) DNA/RNA polymerase, allowing for robust amplification at a constant temperature and production of up to 10^9^ target gene copies within a single hour (19, 20). LAMP has emerged as a promising diagnostic platform for POC detection of clinically important pathogens, such as *Mycobacterium tuberculosis*, human immunodeficiency virus (HIV), and human influenza virus (21–23). However, the specificity of LAMP remains a challenge, primarily attributed to dependence on nonspecific detection methodologies including intercalating fluorescent dyes, colorimetric indicators, and turbidity measurements for signal interpretation (24).

Owing to its high specificity, restriction endonuclease-mediated real-time LAMP has become an attractive basis for various probe-based diagnostic platforms for clinical applications. In previous studies, the restriction endonuclease-mediated real-time fluorescence (REF) approach was verified as a promising tool for specific analysis of LAMP products (25, 26). In the present study, inspired by the basic mechanisms of REF and LAMP, we developed a sequence-specific, easy-to-use HBV-RT-LAMP diagnostic system (HBV-RT-LAMP). HBV pgRNA was selected as the target gene for which the LAMP primer sets were designed, and the probe was designed based on the primer sets. The loop primer LF was modified with a restriction endonuclease recognition sequence (RERS) at the 5′ end and renamed LF*. The RERS can be specifically recognized by the Nb.*BsrDI* enzyme. LF* was labeled at the 5′ end with a reporter dye (FAM) and in the middle with a corresponding dark quencher (BHQ1). Initially, the emission from FAM is quenched because BHQ1 is in close proximity. During the LAMP stage, the double-stranded terminal sequence (containing the RERS) is cleaved by the Nb.*BsrDI* enzyme, resulting in the generation of a fluorescence signal. In addition, a gold nanoparticle-based lateral flow biosensor (AuNPs-LFB), which is a preeminent paper-based POC testing tool, was used for visual readout of the HBV-RT-LAMP products (27–29). For AuNPs-LFB interpretation, FIP and LF primers were labeled at the 5′ end with FAM and biotin, respectively, and the HBV-RT-LAMP products were simultaneously labeled with biotin and FAM for visual detection. Overall, this novel detection system is expeditious, straightforward, and accurate, obviating the necessity for specialized equipment. The feasibility of our assay was validated using clinical specimens from patients with chronic HBV.

## RESULTS

### HBV-RT-LAMP assay system overview

**Fig 1:**
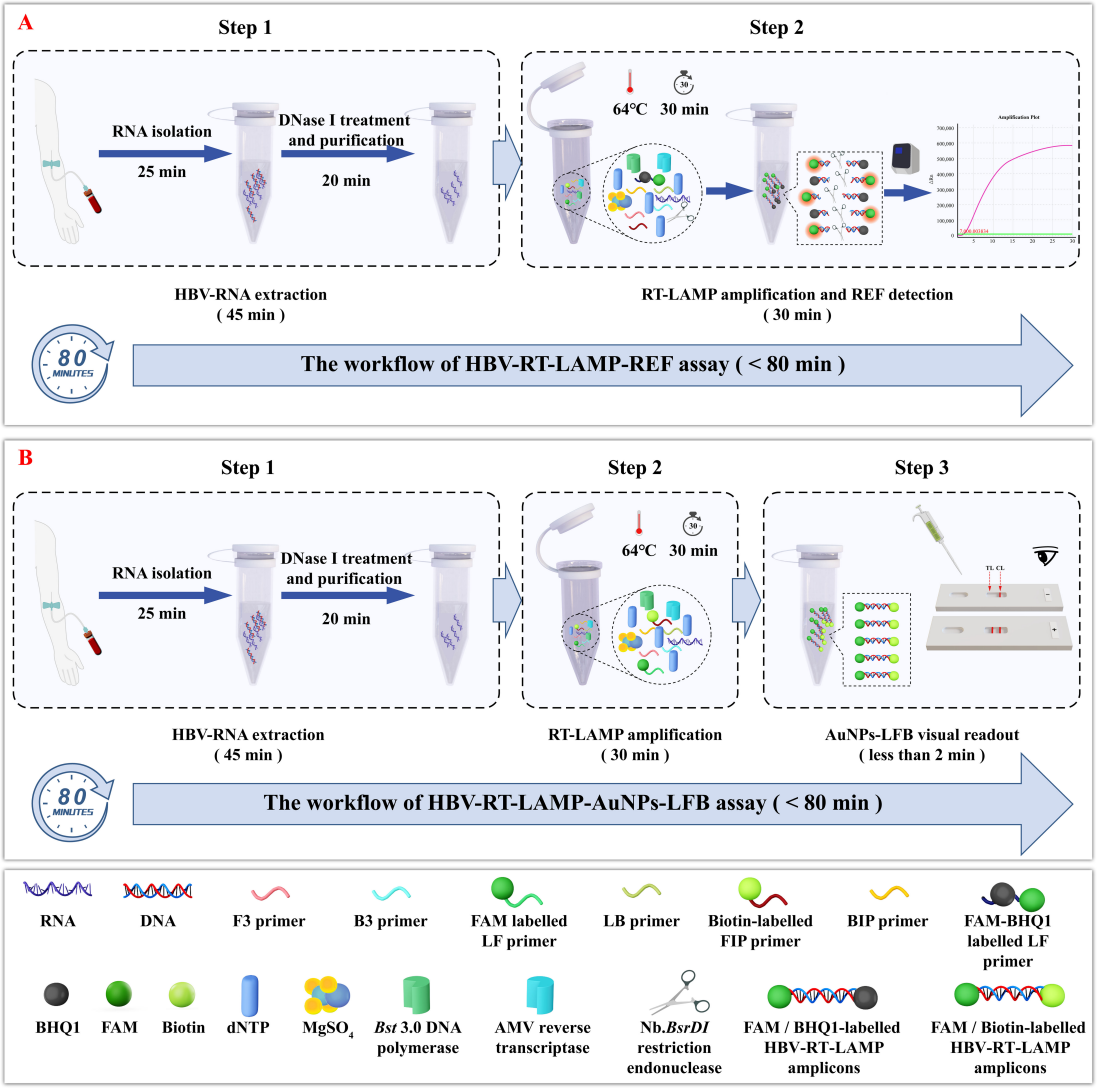
Outline of the HBV-RT-LAMP assay workflow. (**A**) HBV-RT-LAMP-REF assay. The entire workflow employs the following closely linked steps: the HBV RNA was extracted and treated with DNase I (step 1), RT-LAMP amplification, and REF interpretation (step 2). (**B**) HBV-RT-LAMP-AuNPs-LFB assay. The entire workflow employs three closely linked steps: the HBV RNA was extracted and treated with DNase I (step 1), RT-LAMP amplification (step 2), and AuNPs-LFB visual readout (step 3). The whole diagnostic procedure can be completed within 80 min.

The underlying principles and workflow of the HBV-RT-LAMP assay are shown in Fig. 1. In brief, HBV RNA was extracted and treated with DNase I as previously described (Fig. 1A and B, step 1). Next, the target gene was amplified via RT-LAMP for 30 min at a constant temperature of 64°C (Fig. 1A and B**,** step 2). The results were analyzed simultaneously using REF (Fig. 1A**,** step 2) and visual AuNPs-LFB technologies (Fig. 1B**,** step 3). The whole detection process was completed within 80 min.

**Fig 2:**
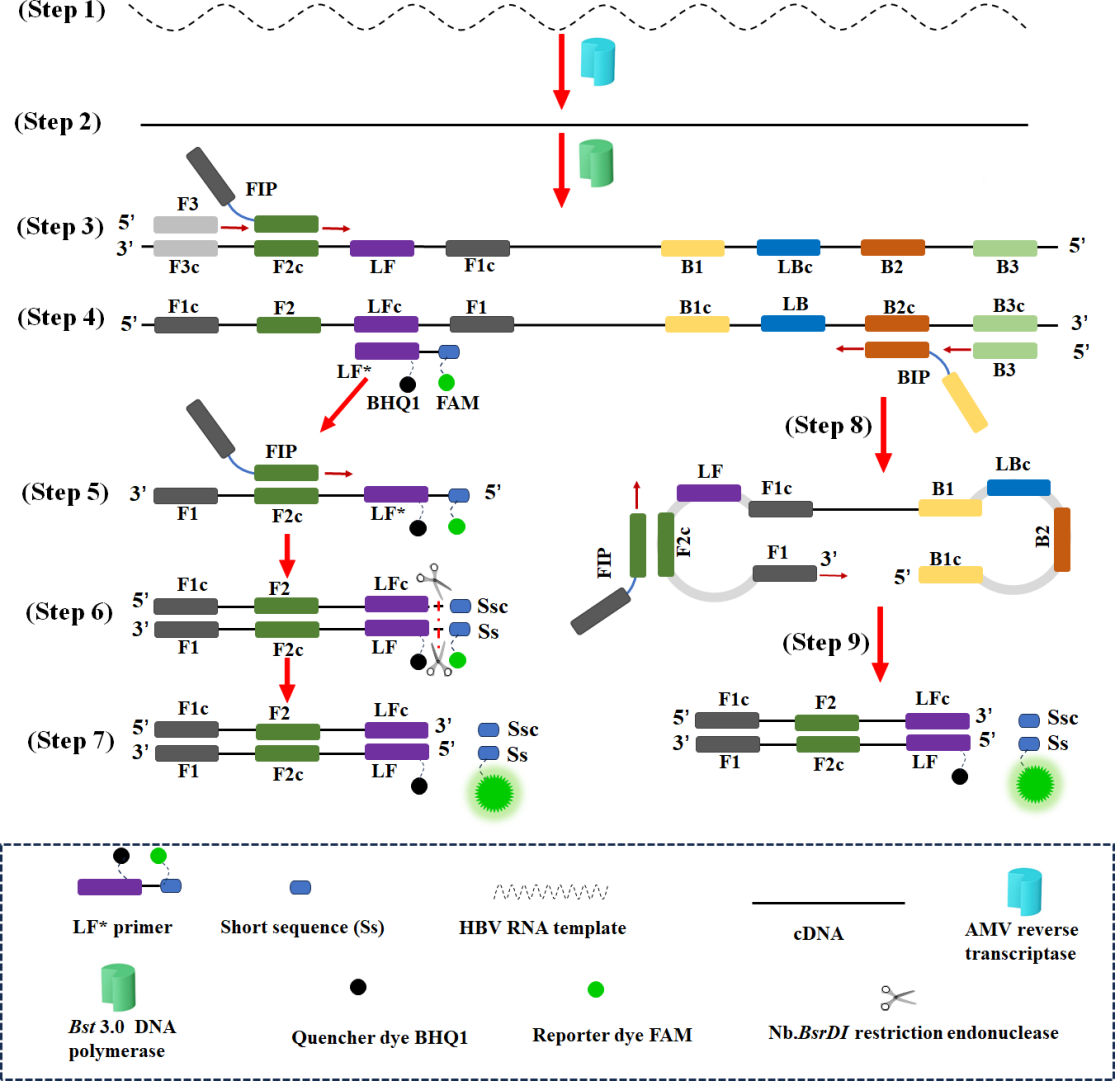
The schematic reaction mechanism of HBV-RT-LAMP-REF assay. Step 1: the HBV-RNA templates were converted to cDNA by AMV reverse transcriptase. Step 2–5: the cDNA strands as templates were amplified in the LAMP reaction system. Step 6: the short sequences were recognized and cleaved by the Nb. *BsrDI* restriction endonuclease. Step 7–9: the fluorescein groups (FAM) and the dark quenchers (BHQ1) were separated, and the fluorescent signal was detected by a real‐time fluorescence detector.

The schematic mechanism of the HBV-RT-LAMP-REF assay is presented in Fig. 2. The diagnostic system integrates HBV RNA reverse transcription, LAMP, restriction endonuclease digestion, and real-time fluorescence analysis into a one-pot reaction mixture. The loop primer LF was modified with a short sequence at the 5′ end (5′-GCAATGNN-3′; N: A, G, C, and T), and the modified LF was termed LF^*^. The short sequence was designed to be recognized by the Nb.*BsrDI* restriction endonuclease, and an additional base (T) was added to the 5′ end of LF^*^ to protect the recognition site. Then, LF^*^ was labeled at the 5′ end with a reporter dye (FAM) and in the middle with a corresponding dark quencher (BHQ1). A set of LAMP primers, including F3, B3, FIP, BIP, LF^*^, and LB, was used. Avian myeloblastosis virus (AMV) reverse transcriptase converted the HBV RNA to cDNA, which then served as the initial template for the subsequent LAMP reaction (Fig. 2**,** steps 1 and 2). FIP hybridized to F2c of the target sequence and initiated complementary strand synthesis, and F3 hybridized to F3c of the target gene to initiate a strand displacement reaction through *Bst* 3.0 strand displacement polymerase, producing a signal strand (Fig. 2**,** step 3). The newly single-stranded DNA acted as a template for LF^*^-primed strand displacement DNA synthesis; moreover, the BIP and B3 primers initiated a new DNA synthesis (Fig. 2, step 4). Next, the LF^*^ strand served as the template for sequence extension through the FIP primer (Fig. 2, step 5). The new double-stranded terminal sequence, containing short sequences and their complementary sequences, was cleaved by the Nb.*BsrDI* restriction endonuclease (Fig. 2, step 6), resulting in FAM fluorescence signal emission (Fig. 2, step 7). In addition, the BIP sequence formed a dumbbell-shaped amplicon that can rapidly convert to a stem-loop form sequence by self-primed synthesis, serving as a DNA template for the next amplification cycle (Fig. 2, step 8). The subsequent exponential amplification products were also cleaved by Nb.*BsrDI*, resulting in exponential FAM reporter dye fluorescence and enabling real-time fluorescence detection (Fig. 2, step 9).

**Fig 3:**
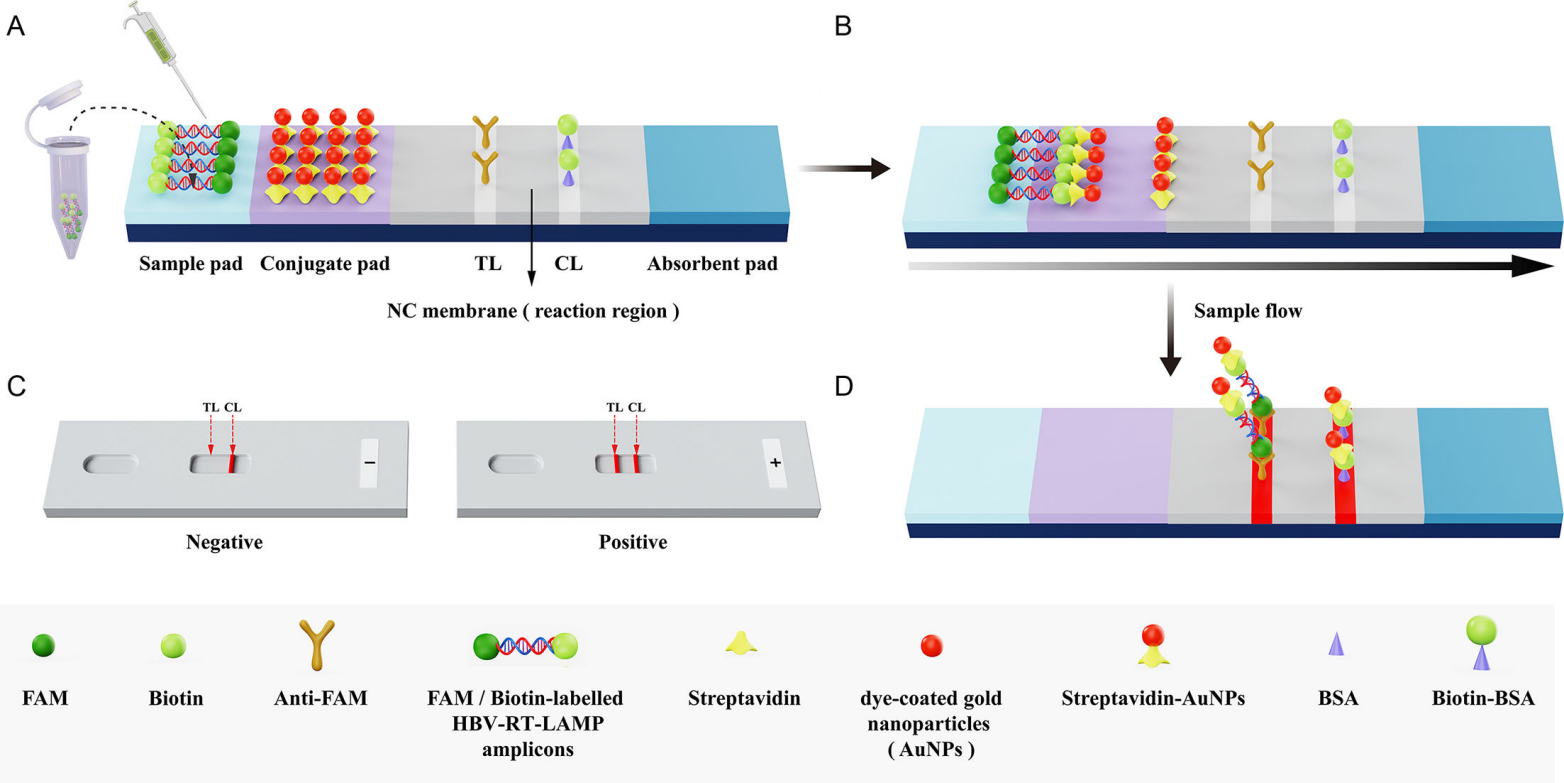
Schematic mechanism of AuNPs-based biosensor for detection of HBV-RT-LAMP amplicons. (**A**) HBV-RT-LAMP amplicons (1 µL) and running buffer (PBS buffer, 100 µL) were dripped to the sample pad. (**B**) The running buffer-containing LAMP amplicons were moved along the AuNPs-LFB biosensor through capillary action, and then the FAM/biotin-labeled HBV-RT-LAMP products were integrated with streptavidin-AuNPs. (**C**) The FAM/biotin-HBV-RT-LAMP-streptavidin-AuNPs complexes were fixed by anti-FAM at the TL region, and the residual streptavidin-AuNPs were captured by biotin-BSA at the CL region. (**D**) Interpretation of the HBV-RT-LAMP-AuNPs-LFB assay. For a positive result, both the CL and TL turned red; negative results are indicated when only the CL band appears on the biosensor.

The schematic diagram of the mechanism by which the AuNPs-LFB device detects HBV-RT-LAMP products is depicted in Fig. 3. In brief, HBV-RT-LAMP amplicons (1 µL) and running buffer (PBS buffer, 100 µL) were dripped onto the sample pad (Fig. 3A). The running buffer, containing the LAMP amplicons, moved along the AuNPs-LFB device through capillary action. The streptavidin-functionalized gold nanoparticles (SA-AuNPs) were rehydrated via running buffer, and then the FAM/biotin-labeled HBV-RT-LAMP products were integrated with the SA-AuNPs (Fig. 3B). At the detection pad, the FAM/biotin-labeled HBV-RT-LAMP–SA-AuNP complexes were fixed by anti-FAM at the test line (TL) region, and the residual SA-AuNPs were captured by biotin-BSA at the control line (CL) region (Fig. 3C). The readout of the HBV-RT-LAMP-AuNPs-LFB results is presented in Fig. 3D. A positive result was defined as both the CL and TL turning red; a negative result, as only the CL turning red.

### Validity of the LAMP primers for HBV-RT-LAMP assay

**Fig 4:**
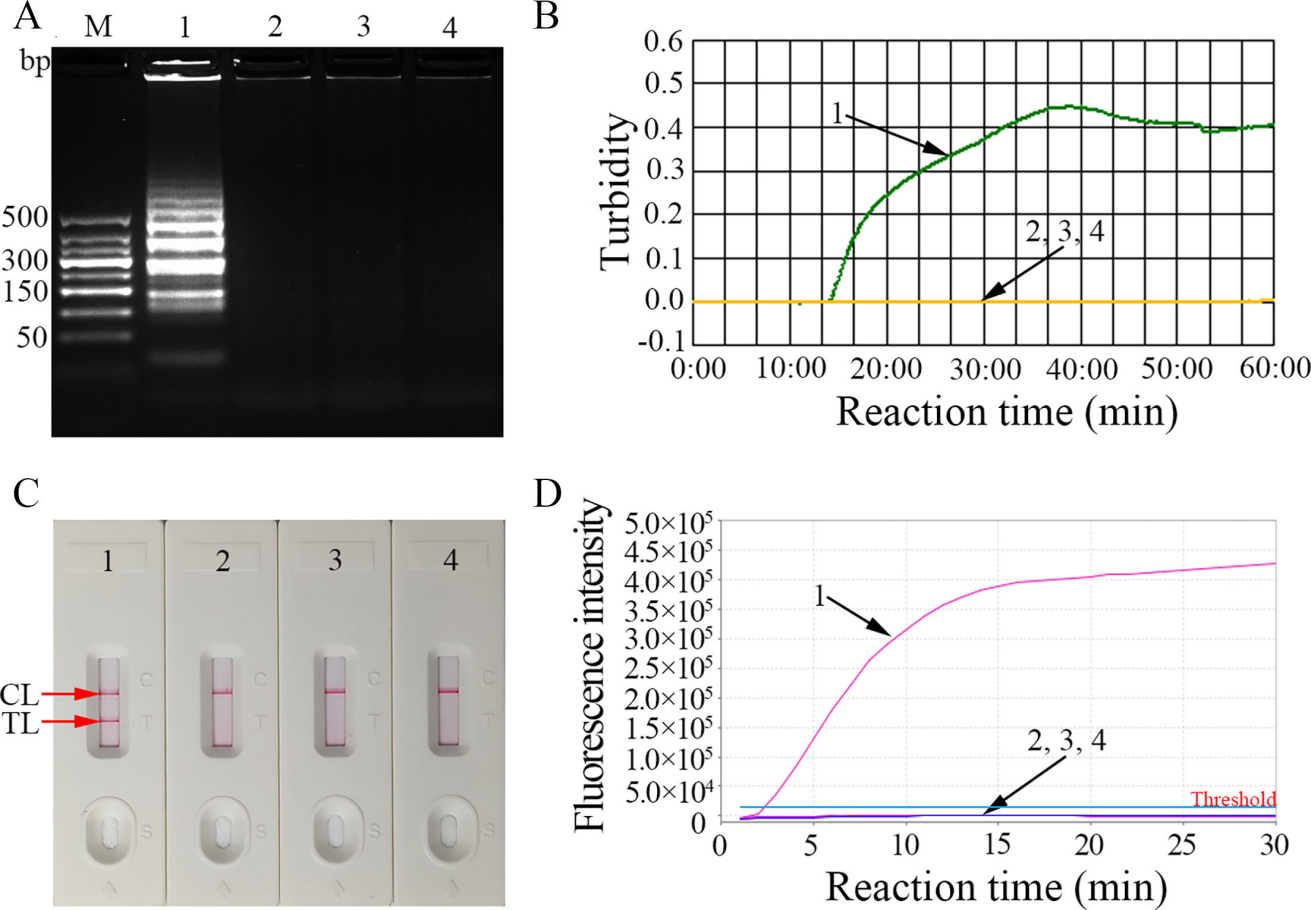
Confirmation of the HBV-RT-LAMP diagnostic system. The HBV-RT-LAMP diagnostic system was verified simultaneously through (**A**) 2% agarose gel electrophoresis, (**B**) real-time turbidity, (**C**) AuNPs-LFB biosensor, and (**D**) REF methods. Templates of A1/B1/C1/D1-A4/B4/C4/D1 were the HBV-RNA standard substance, HBV-DNA standard substance (treated with DNase I), HCV, and ddH_2_O (blank control), respectively.

To validate the LAMP primer set, LAMP reaction mixtures were incubated at a constant temperature of 65°C for 1 h. Next, the LAMP amplicons were analyzed simultaneously with 2% agarose gel electrophoresis, real-time turbidimetry, REF, and AuNPs-LFB assay. The templates from the HBV RNA standards were significantly amplified, while no amplification from the templates of HBV DNA (treated with DNase I), hepatitis C virus (HCV), or blank control (ddH_2_O) was observed (Fig. 4A through D). These data indicated that the LAMP primer set targeting HBV pgRNA was valid for application in the HBV-RT-LAMP assay.

### Optimal reaction temperature for HBV-RT-LAMP assay

Temperature optimization of the LAMP pre-amplification phase in the HBV-RT-LAMP diagnostic system was systematically investigated using HBV RNA standards (1.0 × 10^4^ copies/mL) across a temperature gradient spanning 56°C–66°C (2°C increments). The outcomes of LAMP were monitored with a real-time turbidimeter, and optimal amplification efficiency of HBV RNA was observed at 64°C (Fig. S2a through f).

### Sensitivity of HBV-RT-LAMP assay

**Fig 5:**
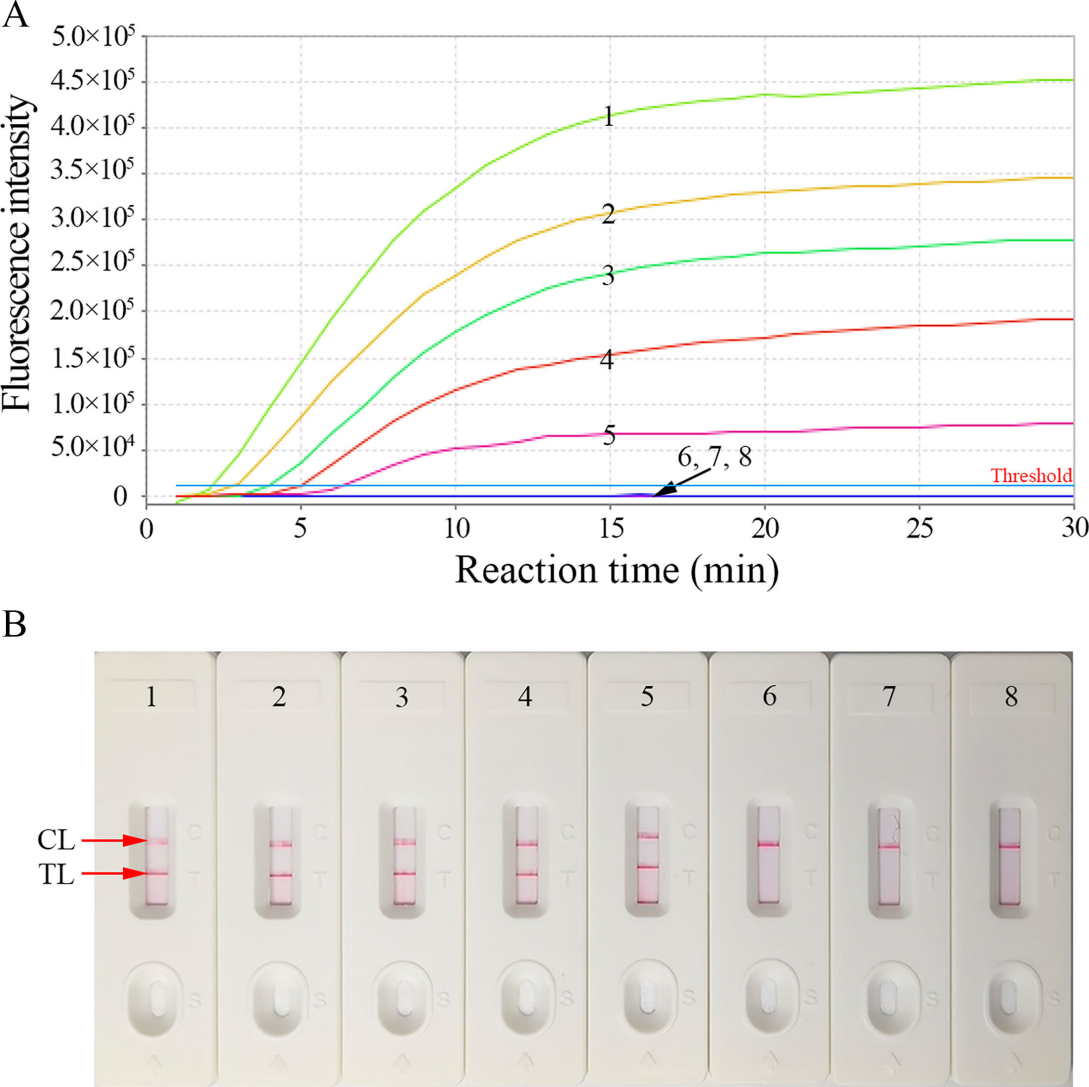
Sensitivity of the HBV-RT-LAMP diagnostic system. (**A**) REF and (**B**) AuNPs-LFB approaches were simultaneously used to analyze the HBV-RT-LAMP amplicons. 1–8 represented the HBV-RNA standard substance concentrations 1 × 10^5^, 1 × 10^4^, 1 × 10^3^, 1 × 10^2^, 50, 10, and 1 copies/mL, and ddH_2_O, respectively. The LoD of the HBV-RT-LAMP diagnostic system was 50 copies/mL.

To analyze the sensitivity of our HBV-RT-LAMP assay, serial dilutions of HBV RNA standards (1.0 × 10^5^ to 1 copy/mL) were systematically evaluated at the optimal reaction temperature (64°C) for 1 h. The results were validated using REF (Fig. 5A) and AuNPs-LFB (Fig. 5B). The limit of detection (LoD) of our assay was 50 copies/mL (Fig. 5A and B). Strikingly, AuNPs-LFB results demonstrated 100% concordance with REF-derived results across all detectable concentrations, confirming the assay’s reliability for both instrumental fluorescence and naked-eye diagnostics.

### Optimal reaction time for HBV-RT-LAMP assay

To optimize the amplification time of our HBV-RT-LAMP assay, multiple reaction times (20, 25, 30, 35, and 40 min) for LAMP were tested at the established optimal temperature (64°C). The outcomes were validated through REF and AuNPs-LFB. The LoD of 50 copies/mL of the HBV RNA standard was detected via visual AuNPs-LFB at 30 min (Fig. S3B), and a stable and robust REF signal also appeared within 30 min (Fig. S3A).

### Specificity of HBV-RT-LAMP assay

**Fig 6:**
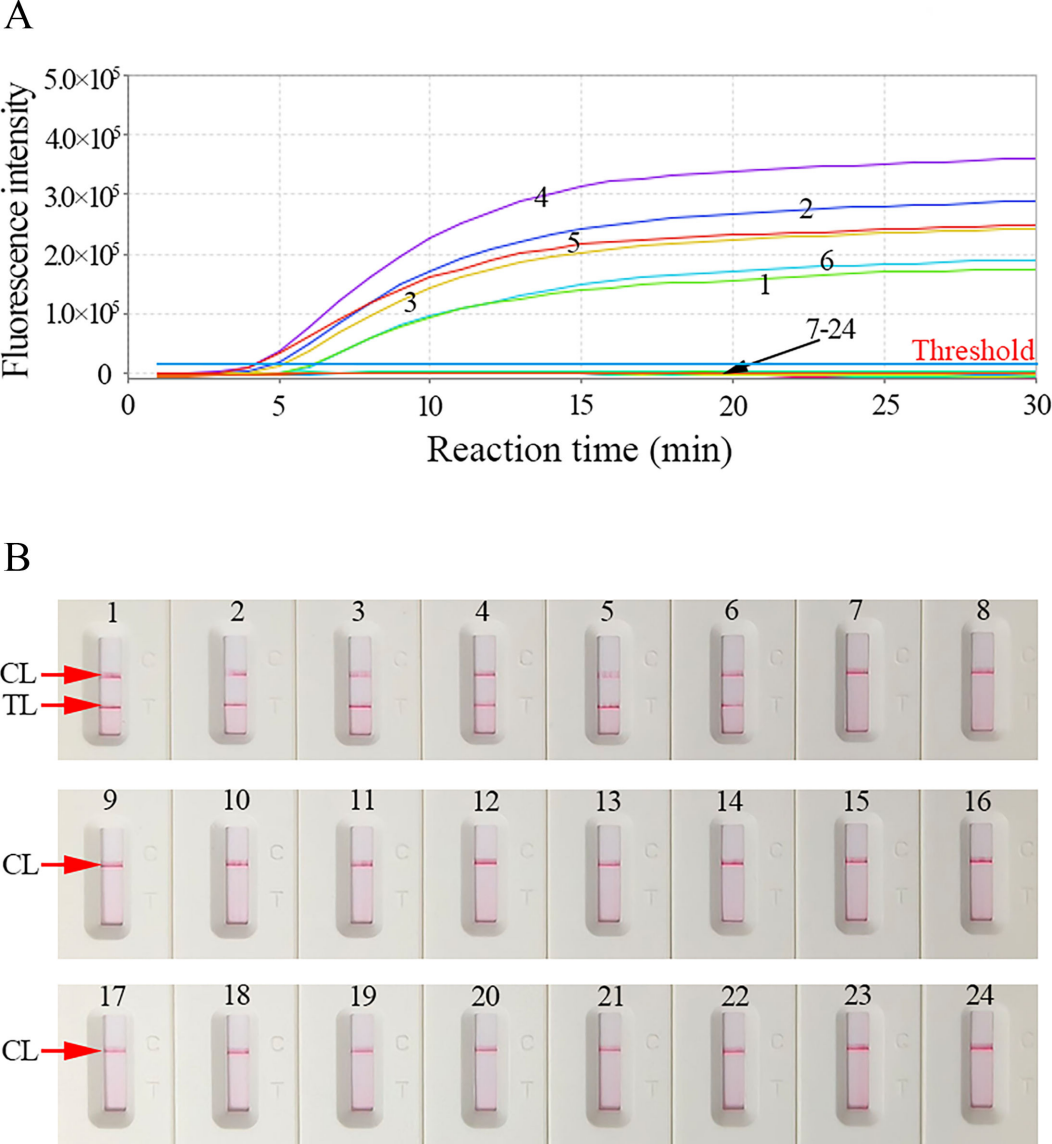
Specificity analysis of HBV-RT-LAMP diagnostic system with different strains. The LAMP reactions were performed using different nucleic acid templates, and each of the amplification products was analyzed through (**A**) REF and (**B**) AuNPs-LFB approaches. A1/B1: HBV-RNA standard substance; A2/B2-A6/B6: five HBV-RNA positive clinical serums (confirmed with HBV-RNA-qPCR); A7/B7-A24/B24: HBV-DNA (treated with DNase I), HCV, HIV, Epstein-Barr virus, human enterovirus EV71, Coxsackie virus CAV16, influenza A virus, influenza B virus, human papillomavirus, parainfluenza virus, Adenoviridae, herpes zoster virus, measles virus, human rhinovirus, *Haemophilus influenzae*, *Mycobacterium tuberculosis*, *Staphylococcus aureus*, and ddH_2_O, respectively.

High specificity is a fundamental requirement for the clinical application of nucleic acid amplification-based diagnostic assays. Here, the specificity of our assay was evaluated using HBV RNA standard, HBV RNA positive clinical serums (confirmed by HBV RNA real-time TaqMan PCR), HBV DNA standards (treated with DNase I), and other microbial nucleic acid templates (Table 1). The HBV-RT-LAMP assay protocol was conducted as described above, and the results were analyzed with REF and AuNPs-LFB. As shown in Fig. 6, only the HBV RNA templates exhibited a markedly elevated fluorescence signal (Fig. 6A), and the TL and CL were simultaneously generated at the AuNPs-LFB detection zone (Fig. 6B). HBV DNA and non-HBV microbes presented negative outcomes (Fig. 6A and B). These results indicated that our assay could identify HBV-RNA with high specificity. No cross-reactions were observed.

**TABLE 1:** Microbial strains used in the current study

No.	Strains/templates	Source of strains^*a*^	No. of strains	HBV-RT-LAMP assay^*b*^	
				AuNPs-LFB	REF
1	HBV-RNA (standard substance)	National Institutes for Food and Drug Control	1	P	P
2	HBV-RNA (clinical samples)	Second GZUTCM	5	P	P
3	HBV-DNA (treated with DNase I)	National Institutes for Food and Drug Control	1	N	N
4	HCV	Second GZUTCM	1	N	N
5	HIV	GZCCL	1	N	N
6	Epstein-Barr virus	Second GZUTCM	1	N	N
7	Human enterovirus EV71	GZCDC	1	N	N
8	Coxsackie virus CAV16	GZCDC	1	N	N
9	Influenza A virus	GZCCL	1	N	N
10	Influenza B virus	GZCCL	1	N	N
11	Human papillomavirus	GZCCL	1	N	N
12	Parainfluenza virus	GZCDC	1	N	N
13	Adenoviridae	GZCCL	1	N	N
14	Herpes zoster virus	Second GZUTCM	1	N	N
15	Measles virus	GZCDC	1	N	N
16	Human rhinovirus	GZCDC	1	N	N
17	*Haemophilus influenzae*	ATCC49247	1	N	N
18	*Mycobacterium tuberculosis*	GZCDC	1	N	N
19	*Staphylococcus aureus*	Second GZUTCM	1	N	N

^*a*^ Second GZUTCM, the Second Affiliated Hospital, Guizhou University of Traditional Chinese Medicine; GZCDC, Guizhou Provincial Center for Disease Control and Prevention; GZCCL, Guizhou Provincial Center for Clinical Laboratory; ATCC, American Type Culture Collection.

^*b*^ P, positive; N, negative.

### Evaluating the feasibility of the HBV-RT-LAMP assay using clinical samples

To further evaluate the HBV-RT-LAMP platform as a valuable tool for HBV RNA identification, 40 serum specimens from CHB patients (undergoing antiviral treatment) and 16 serum specimens from healthy donors were tested simultaneously using the HBV-RT-LAMP diagnostic system, HBV-RNA-qPCR, and HBV-DNA-qPCR. All 40 CHB samples were correctly identified as positive via HBV-RNA-qPCR, and these results were in accordance with those of our HBV-RT-LAMP assay. However, the HBV-DNA qPCR results only identified 37 CHB samples as positive. All three methods correctly identified the 16 serum specimens from healthy donors as negative (Table 2; Table S1; Fig. S4 and S5). These data indicated that the HBV-RT-LAMP diagnostic system functioned as an advanced and convenient diagnostic tool for identifying CHB infection, especially in the antiviral treatment stage.

**TABLE 2:** Comparison of HBV-RT-LAMP diagnostic system, HBV-RNA-RT-qPCR, and HBV-DNA-RT-qPCR methods to detect chronic HBV infection in clinical samples

Detection assay	Results	Reference method (HBV-RNA-qPCR)^*a*^		True positive rate (%)	True negative rate (%)
		+	–		
HBV-DNA-qPCR	+	37	0	92.5%	100.00%
	–	3	16		
HBV-RT-LAMP diagnostic system	+	40	0	100.00%	100.00%
	–	0	16		

^*a*^ +, positive; –, negative.

## DISCUSSION

CHB imposes a major global health burden affecting more than two billion individuals worldwide, and 15%–40% of chronic HBV carriers progress to cirrhosis and HCC (30, 31). Accurate, rapid, easy, and visual diagnostic methods are critical for effective treatment and prevention of progression to terminal hepatic complications. In the current study, we developed a novel, pgRNA-targeting HBV-RT-LAMP diagnostic platform that integrates probe-based LAMP with REF or AuNPs-LFB for highly specific, sensitive, and rapid visual identification of serum HBV RNA.

Traditionally, serological and molecular markers, including HBsAg and HBV DNA, were used to identify chronic HBV infection and determine the timing of treatment and drug withdrawal (12). The clearance of serum HBsAg is regarded as the key indicator for the “functional cure” of CHB (32, 33). However, HBV DNA can be integrated into the genome of HBV-infected hepatocytes, and CHB patients may sustain low expression levels of HBsAg in peripheral blood (34, 35). In this case, HBsAg may not accurately reflect cccDNA activity in hepatocytes. Currently, serum HBV DNA levels are used to assess the efficacy of antiviral therapy (36). Nevertheless, concentrations of HBV DNA that fall below the detection limit only indicate that the reverse transcription of HBV DNA is suppressed and therefore should not serve as the criterion for drug withdrawal (37). In contrast, serum pgRNA is produced only by cccDNA and directly reflects hepatocytic cccDNA activity (38). Hence, it is useful for identifying the presence of serum HBV RNA during antiviral therapy. In the present study, our HBV-RT-LAMP diagnostic platform specifically targeted serum-derived pgRNA. The clinical feasibility of our assay was validated using serum samples obtained from patients with chronic HBV infection, and the results were compared with those of qPCR assays for both HBV DNA and HBV RNA detection. Both our assay and HBV-RNA-qPCR correctly identified all HBV RNA-positive samples (40/40). However, HBV-DNA-qPCR only identified 37 out of 40 HBV RNA-positive samples. In this study, all 40 CHB patients were treated with NAs therapy. At this stage, serum HBV-DNA could not serve as a reliable biomarker because a level that falls below the detection limit does not necessarily imply the elimination of the virus; it only indicates that its reverse transcription is suppressed (39). Hence, the HBV-DNA-qPCR results only identified 37 CHB samples as positive, and all 40 CHB samples were correctly identified as positive via HBV-RNA-qPCR and our HBV-RT-LAMP assay. These data preliminarily indicated that the HBV-RT-LAMP diagnostic system functioned as an advanced and convenient diagnostic tool for identifying CHB infection, especially in the antiviral treatment stage. However, we just collected a modest clinical sample size (40 CHB, 16 healthy controls) for testing the feasibility of our assay. To more comprehensively evaluate our assay, it is necessary to test a wider variety of HBV genotype samples from CHB patients with low viral loads during antiviral therapy, and the clinical utility of our assay requires evaluation in real-world, low-resource settings.

Currently, PCR-based techniques are the mainstay for serum HBV RNA identification in CHB patients. Nguyen et al. applied RT-PCR to identify pgRNA with an LoD as low as 100 copies/mL (13). Gao et al. used a one-step quantitative reverse-transcription PCR assay to detect pgRNA with an LoD of 100 copies/reaction (40). However, these methods are inaccessible and unaffordable in resource-limited regions because they require high-precision thermal cyclers, robust laboratory infrastructure, and trained personnel. Traditional isothermal amplification techniques, including LAMP and recombinase polymerase amplification, enable nucleic acid amplification at a fixed temperature without specific facilities. Despite their advantages, these methods suffer from intrinsic limitations in analytical sensitivity and specificity (24). Zhu et al. integrated CRISPR/Cas13a with reverse transcription-multienzyme isothermal rapid amplification for HBV RNA identification with a detection sensitivity of 10^3^ copies/mL (41), but this approach requires a fluorescence detector. In this study, we developed a novel, pgRNA-targeting HBV-RT-LAMP diagnostic platform that integrates probe-based LAMP with REF/AuNPs-LFB for highly specific, sensitive, and rapid visual identification of serum HBV-pgRNA. The results can be visually read out with AuNPs-LFB or a fluorescence detector. Our assay demonstrated a 50 copies/mL LoD and had no cross-reactions with any other pathogens (Fig. 5 and 6). The detection process, including HBV RNA extraction (45 min), RT-LAMP (30 min), and results interpretation (AuNPs-LFB, less than 2 min), was performed within 80 min. These data confirmed that our novel assay can effectively and accurately identify HBV RNA.

In this study, the HBV pgRNA was robustly amplified and specifically identified through *Bst* 3.0 DNA polymerase and LAMP primer-probe modification. For REF detection, the loop primer LF was connected to a short Nb.*BsrDI* RERS and labeled with a reporter dye (FAM) and corresponding dark quencher (BHQ1) at the 5′ end and in the middle, respectively. During the amplification process, the restriction endonuclease site was recognized and cleaved by the Nb.*BsrDI* enzyme, and the FAM signal was emitted and detected by the fluorescence detector. For AuNPs-LFB visual identification, the HBV pgRNA FIP^#^ and LF^#^ primers were labeled at the 5′ end with FAM and biotin, respectively. Upon the LAMP reaction, the HBV-RT-LAMP products were simultaneously labeled with FAM and biotin. A positive outcome was observed if the FAM/biotin-labeled HBV-RT-LAMP products were captured by anti-FAM at the TL, and the SA-AuNPs were fixed with biotin-BSA at the CL, resulting in two visible crimson lines (TL and CL) appearing simultaneously on the AuNPs-LFB strip. A negative outcome was recorded if only the CL line was observed. The optimal conditions for the RT-LAMP pre-amplification stage were determined to be 64°C and 30 min. The approximate total cost of a single HBV-RT-LAMP assay, including LAMP isothermal reagent (approximately US $0.5), Nb.*BsrDI* restriction endonuclease (approximately US $0.5), AuNPs-LFB (approximately US $2.0), and other reagents and materials (approximately US $1.0), was not to exceed US $4.0 per reaction, which is cheaper than that of traditional real-time PCR testing (approximately US $7.0 per reaction) (42).

Previously, we also combined LAMP reaction with AuNPs-LFB for the identification of HIV type one (HIV-1) and severe acute respiratory syndrome coronavirus 2 with an LoD as low as 20 copies per reaction (22, 43). In addition, we also used LAMP-AuNPs-LFB platform for the detection of HBV-DNA with an LoD as low as 7.5 IU per test (44). However, these methods required opening the LAMP reaction tube for AuNPs-LFB interpretation, which increased the risk of carryover contamination. In the current study, the REF method was successfully designed for LAMP product readout, which can achieve the detection process in one tube. More importantly, the HBV-pgRNA was selected as the target gene for our HBV-RT-LAMP development, which is a more accurate evaluation of chronic HBV infection status, especially during the period of antiviral drug treatment.

Our study had some limitations. First, detecting HBV RNA presents a challenge because the size of the HBV pgRNA sequence, which is 1.1 times the size of the HBV genome after reverse transcription and covers the entire HBV relaxed circular DNA sequence (45). In order to facilitate effective HBV pgRNA detection, using DNase I to pre-digest HBV DNA from the extracted RNA mixture is an effective way (14, 46). However, this multi-step process requires much more time, and the HBV RNA template extraction process could be refined in further studies. Second, our assay can only be used for qualitative HBV RNA identification, especially using AuNPs-LFB biosensor for visual interpretation of HBV-RT-LAMP products. It is noteworthy that several studies have employed LAMP for semi-quantitative or quantitative detection. Yu et al. combined LAMP and image processing with the hue-saturation-value color model for semi-quantitative detection of African swine fever virus (47), and Papadakis et al. developed a device for performing real-time colorimetric LAMP combining the accuracy of lab-based quantitative analysis with the simplicity of POC testing (48). Establishing a quantitative method for HBV-RT-LAMP represents an important direction for future research. Third, analyzing the HBV-pgRNA-LAMP products through the AuNPs-LFB platform required opening the reaction tube, which increased the risk of carryover contamination. In our laboratory, the immediate application of a nucleic acid scavenger following each AuNPs-LFB test has proven to be an effective measure for preventing nucleic acid contamination. For improved adaptability in clinical settings, the HBV-RT-LAMP diagnostic system should be refined through the development of a sealed LAMP-AuNP-LFB device, which prevents aerosol contamination by eliminating the tube-opening procedure.

In conclusion, we successfully integrated probe-based LAMP with REF or AuNPs-LFB and developed a novel HBV-RT-LAMP detection system for specific, sensitive, rapid, and visual identification of HBV pgRNA. Our assay demonstrated a 50 copy/mL LoD and had no cross-reactions with any other pathogens. The whole detection process, including HBV RNA extraction (45 min), LAMP (30 min), and results interpretation (AuNPs-LFB, less than 2 min), was performed within 80 min, with no need for specialized devices. Hence, our assay can potentially serve as a useful POC diagnostic tool for more accurate and convenient evaluation of chronic HBV infection status and antiviral drug efficacy.

## MATERIALS AND METHODS

### Materials and instruments

*Bst* 3.0 DNA polymerase, AMV reverse transcriptase, Nb.*BsrDI* restriction endonuclease, deoxynucleotide (dNTP) mix, and MgSO_4_ solution were obtained from New England Biolabs (Ipswich, USA). AuNPs-LFB materials, including nitrocellulose (NC) membranes, backing cards, sample pads, conjugate pads, and absorbent pads, were manufactured by Hui-De-Xing Biotech Co., Ltd. (Tianjing, China) according to our design specifications. Streptavidin-coated gold nanoparticles (crimson red) were provided by Bangs Laboratories, Inc. (Indiana, USA). Biotinylated bovine serum albumin (biotin-BSA) and anti-fluorescein antibody (anti-FAM) were obtained from Abcam Co., Ltd. (Shanghai, China). EasyPure Viral RNA Kit was purchased from TransGen Biotech Co., Ltd. Co., Ltd. (Beijing, China). A real-time turbidimeter (LA-500) was sourced from Eiken Chemical Co., Ltd. (Tokyo, Japan). A ChemiDoc MP imaging system was obtained from Bio-Rad Co., Ltd. (California, USA).

### Standard panels and clinical samples

The National Standard for HBV RNA detection (Batch No. 340023-202001) was obtained from the National Institutes for Food and Drug Control. A total of 40 serum samples were collected from CHB patients undergoing antiviral treatment. For the negative control group, 16 serum specimens were obtained from healthy donors with no history of HBV infection. HBV RNA was extracted using the EasyPure Viral RNA Kit (TransGen Biotech, China) in accordance with the manufacturer’s instructions and treated with DNase I (New England Biolabs, USA) at 37°C for 10 min. Finally, the RNA was purified with Spin RNA Cleanup Kit (New England Biolabs, USA).

### LAMP primer design

The LAMP primers were designed using the online software Primer Explorer Version 5.0 (https://primerexplorer.eiken.co.jp/lampv5/index.html) and PRIMER PREMIER 5.0 in accordance with the mechanism of LAMP targeting HBV pgRNA (GenBank accession no. LR745632.1). A set of LAMP primers containing two outer primers (F3 and B3), two inner primers (FIP and BIP), and two loop primers (LF and LB) was designed. The specificity of the HBV-RT-LAMP primers was validated using the National Center for Biotechnology Information basic local alignment search tool. The locations of the LAMP primers are shown in Fig. S1, and the details of primer sequences and modifications are shown in Table 3. All oligonucleotides were synthesized and purified via high-performance liquid chromatography by TsingKe Biotech Co., Ltd. (Beijing, China).

**TABLE 3:** HBV-RT-LAMP primers used in this study

Primer name^*a*^	Sequence and modifications^*b*^	Length^*c*^	Gene
F3	5′-TGACACGTATAAAGAATTTGGAG-3′	23 nt	pgRNA
B3	5′-CATCAACTCACCCCAACA-3′	18 nt	
FIP	5′-GTCGAGAAGATCTCGAATAGAAGGACTTCTGTGGAGTTACTCTCT-3′	45 mer	
FIP^#^	5′-FAM-GTCGAGAAGATCTCGAATAGAAGGACTTCTGTGGAGTTACTCTC-3′	45 mer	
BIP	5′-TCTGCTCTGTATCGGGAGGCCACAATAGCTTGCCTGAGA-3′	39 nt	
LF	5′-AGAAGTCAGAAGGCAAA-3′	17 nt	
LF^#^	5′-Biotin-AGAAGTCAGAAGGCAAA-3′	17 nt	
LF^*^	5′- FAM-TGCAATG-AGAAGT(BHQ1)CAGAAGGCAAA-3′	24 nt	
LB	5′-CATTGTTCACCTCACCATA-3′	19 nt	

^*a*^ FIP^#^, 5′-labeled with FAM; LF^#^, 5′-labeled with biotin, when used for the HBV-RT-LAMP-AuNPs-LFB assay; LF* was added with a short sequence at the 5′ end (5′-GCAATG-3′), which can be recognized by Nb.*BsrDI* restriction endonuclease, and an additional base (T) also be added to the 5′ end of LF* to protect the recognition site. Then, the LF* was labeled at the 5′ end with a reporter dye (FAM) and in the middle with a corresponding dark quencher (BHQ1), when used for the HBV-RT-LAMP-REF assay.

^*b*^ FAM, 6-carboxy-fluorescein; BHQ1, black hole quencher 1.

^*c*^ nt, nucleotide; mer, monomeric unit.

### AuNPs-LFB preparation

The schematic representation of the AuNPs-LFB biosensor is illustrated in Fig. 3. Structurally, the biosensor (measuring 60 × 4 mm) comprises four primary functional components sequentially assembled on a plastic backing: a sample pad, a conjugate pad pre-coated with crimson dye-labeled SA-AuNPs, an NC membrane (reaction region), and an absorbent pad. The NC membrane was patterned with two distinct lines: the TL, immobilized with anti-FAM, and the CL, coated with biotin-BSA. The two lines were spatially resolved at a 5 mm interval to ensure unambiguous signal interpretation. All components were laminated onto a rigid plastic substrate using adhesive backing to ensure structural integrity during operation. The AuNPs-LFB devices were fabricated by Tianjin HuiDeXin Biotech Co., Ltd. (Tianjin, China) in accordance with our proprietary design specifications. The biosensors were stored at room temperature before use.

### Working principle of HBV-RT-LAMP assay

For the HBV-RT-LAMP-REF assay, a one-pot reaction (25 µL) was established, containing 1 µL of *Bst* 3.0 DNA polymerase (8 U), 2.5 µL of 10× reaction buffer, 1 µL of AMV reverse transcriptase (10 U), 3.5 µL of dNTP Mix (10 mM), 1.5 µL of MgSO_4_ (100 mM), 1 µL of Nb.*BsrDI* (10 U), 0.4 µM each of F3 and B3, 1.6 µM each of FIP and BIP, 0.8 µM each of LF^*^ and LB, and 1 µL of nucleic acid templates, with double-distilled water (ddH_2_O) added to yield a total volume of 25 µL. The reaction was performed for 30 cycles using the real-time fluorescence instrument at settings of 65°C and 60 s. Amplification mixtures containing 1 µL of ddH_2_O were used as negative controls.

For the HBV-RT-LAMP-AuNPs-LFB detection, another one-pot reaction (25 µL) was established, containing 1 µL of *Bst* 3.0 DNA/RNA polymerase (8 U), 1 µL of AMV reverse transcriptase (10 U), 2.5 µL of 10× reaction buffer, 3.5 µL of dNTP Mix (10 mM), 1.5 µL of MgSO_4_ (100 mM), 0.4 µM each of F3 and B3, 1.6 µM each of FIP^#^ and BIP, 0.8 µM each of LF^#^ and LB, and 1 µL of nucleic acid templates, with ddH_2_O added to yield a total volume of 25 µL. The reaction tubes were processed at 65°C for 60 min. The LAMP amplicons were monitored using AuNPs-LFB. In addition, the LAMP products were detected using agarose gel electrophoresis and a real-time turbidimeter (LA-500).

### Optimization of HBV-RT-LAMP reaction conditions

Temperature represents a critical parameter in isothermal amplification systems, requiring precise optimization to ensure reaction efficiency. A systematic temperature optimization study was conducted across a gradient spanning 56°C–66°C in 2°C increments using an HBV RNA standard (1.0 × 10^4^ copies/mL) as the template. The amplification process was monitored using a real-time turbidimeter. Subsequently, reaction time optimization was performed by taking measurements at discrete intervals (20, 25, 30, 35, and 40 min) to determine the minimum required amplification stage, using the LoD HBV RNA standard. Readout of the results was conducted simultaneously using AuNPs-LFB and REF methodologies. Each test was replicated in triplicate.

### Sensitivity and specificity of the HBV-RT-LAMP assay

To evaluate the sensitivity of the HBV-RT-LAMP assay, HBV RNA standard material (containing sensitivity reference material) was subjected to a 10-fold serial dilution (from 1.0 × 10^5^ to 1 copys/mL), and the HBV-RT-LAMP reactions were performed under optimal conditions. Reaction products were analyzed in parallel through AuNPs-LFB and REF detection platforms. The LoD of HBV-RT-LAMP was considered the lowest dilution for which all three replicates were positive.

The specificity of our assay was verified through HBV RNA standards, HBV DNA (treated with DNase I), clinical HBV RNA isolates from CHB patient serum, and other microbial nucleic acid templates (Table 1). All the microbial nucleic acid templates used in the current study were extracted using Virus DNA/RNA Extraction Kits or Bacterial Genomic DNA Extraction Kits (Xi'an Tianlong Technology Co., Ltd.; Xi'an, China), and the concentration (>5.0 × 10^4^ copies/mL) was measured with a NanoDrop ND-2000 spectrophotometer (Beijing, China) at A260/280. The nucleic acid templates were stored at −80°C before use. The detection process was performed under the optimized HBV-RT-LAMP reaction conditions with the same set of LAMP primers. All of the amplicons were detected simultaneously with AuNPs-LFB and REF technologies, with ddH_2_O as a blank control. Each test was performed in triplicate.

### Verification of the feasibility of HBV-RT-LAMP assay for clinical samples

To confirm the feasibility of our assay in clinical applications, 40 serum specimens from CHB patients and 16 serum specimens from healthy donors were tested. The virus RNA was isolated using the EasyPure Viral RNA Kit and treated with DNase I at 37°C for 10 min. The RNA was purified with Spin RNA Cleanup Kit (New England Biolabs, USA). Then, the HBV-RT-LAMP assay was performed as described earlier. Additionally, we compared the results of our assay with those obtained using a commercially available real-time TaqMan PCR Kit for HBV RNA and HBV DNA (SanSure Biotech; Changsha, China) on an Applied Biosystems 7500 Real-Time PCR System (Life Technologies; Singapore). Measurements of HBV DNA >5 IU (~30 copies/mL) and HBV RNA >50 copies/mL were regarded as positive, according to the manufacturer’s instructions.

## Data Availability

The original contributions presented in the study are included in the article/supplemental material, and further inquiries can be directed to the corresponding author.

## SUPPLEMENTAL MATERIAL

Supplemental material spectrum.01918-25-s0001.docx Table S1; Fig. S1 to S5.

ASM does not own the copyrights to Supplemental Material that may be linked to, or accessed through, an article. The authors have granted ASM a non-exclusive, world-wide license to publish the Supplemental Material files. Please contact the corresponding author directly for reuse.
