# Supplementary material for: A sequence-specific, nanoparticle-based biosensor platform for rapid and visual identification of serum hepatitis B virus pregenomic RNA in chronic hepatitis B patients
Source: Microbiol Spectr. 2025 Dec 18;14(2):e01918-25. doi: 10.1128/spectrum.01918-25 (PMC12889159; doi:10.1128/spectrum.01918-25)
Supplement: Supplemental material — Table S1; Fig. S1 to S5. [file spectrum.01918-25-s0001.docx]

**SUPPLEMENTAL MATERIAL**

**A sequence-specific, nanoparticle-based biosensor platform for rapid and visual identification of serum hepatitis B virus pregenomic RNA in chronic hepatitis B patients**

Yuanyuan Gu^a,bΔ^, Qi Zhao^a,bΔ^, Yan Tan^c^, Junfei Huang^d^, Yi Wang^e*^, Shijun Li^d*^, Xu Chen^a,b*^

^a^The Second Clinical Medical College, Guizhou University of Traditional Chinese Medicine, Guiyang, Guizhou, 550003, People’s Republic of China

^b^Medical Science Laboratory of Integrative Chinese and Western Medicine, The Second Affiliated Hospital, Guizhou University of Traditional Chinese Medicine, Guiyang, Guizhou, 550003, People’s Republic of China

^c^Clinical Laboratory, Guizhou Provincial Center for Clinical Laboratory, Guiyang, Guizhou 550002, People’s Republic of China

^d^Experimental Center, Guizhou Provincial Centre for Disease Control and Prevention, Guiyang, Guizhou, 550004, People’s Republic of China

^e^Experimental Research Center, Capital Center for Children's Health, Capital Medical University, Capital Institute of Pediatrics, Beijing, 100020, People’s Republic of China

^Δ^Drs. Yuanyuan Gu and Qi Zhao have contributed equally to this work.

^*^Corresponding author:

Xu Chen, E-mail: [xuchen1220@126.com](mailto:xuchen1220@126.com) (Handing the correspondence)

Shijun Li, E-mail: [zjumedjun@163.com](mailto:zjumedjun@163.com)

Yi Wang, E-mail: [wildwolf0101@163.com](mailto:wildwolf0101@163.com)

**Table S1** Comparison of HBV-RT-LAMP diagnostic system, HBV-RNA-qPCR, and HBV-DNA-qPCR methods for assessment of chronic HBV infection in clinical samples

| **Sample No.** | **HBV-RNA-qPCR^a^ (copies/mL)** | **HBV-DNA-qPCR^b^ (copies/mL)** | **HBV-RT-LAMP assay** | |
| --- | --- | --- | --- | --- |
|  |  |  | **REF** | **AuNPs-LFB** |
| Test 1 | 8.94 × 10^2^ | 1.32 × 10^3^ | + | + |
| Test 2 | 3.31 × 10^3^ | 6.84 × 10^3^ | + | + |
| Test 3 | 2.76 × 10^3^ | 5.92 × 10^3^ | + | + |
| Test 4 | 1.98 × 10^3^ | 8.43 × 10^3^ | + | + |
| Test 5 | 7.83 × 10^5^ | 3.16 × 10^6^ | + | + |
| Test 6 | 9.37 × 10^4^ | 1.85 × 10^4^ | + | + |
| Test 7 | 4.81 × 10^5^ | 7.93 × 10^5^ | + | + |
| Test 8 | 2.04 × 10^5^ | 1.91 × 10^6^ | + | + |
| Test 9 | 7.16 × 10^5^ | 6.21 × 10^4^ | + | + |
| Test 10 | 1.37 × 10^3^ | 7.42 × 10^4^ | + | + |
| Test 11 | 5.43 × 10^5^ | 1.69 × 10^6^ | + | + |
| Test 12 | 8.21 × 10^4^ | 4.21 × 10^3^ | + | + |
| Test 13 | 6.02 × 10^5^ | 3.27 × 10^6^ | + | + |
| Test 14 | 9.62 × 10^4^ | 6.15 × 10^3^ | + | + |
| Test 15 | 7.30 × 10^1^ | **—** | + | + |
| Test 16 | 2.83× 10^3^ | 5.82× 10^2^ | + | + |
| Test 17 | 5.47× 10^3^ | 1.13× 10^3^ | + | + |
| Test 18 | 1.24× 10^3^ | 3.28× 102 | + | + |
| Test 19 | 7.12 × 10^3^ | 4.73 × 10^4^ | + | + |
| Test 20 | 5.74 × 10^1^ | **—** | + | + |
| Test 21 | 1.74× 10^4^ | 4.28 × 10^5^ | + | + |
| Test 22 | 1.95 × 10^5^ | 2.74 × 10^6^ | + | + |
| Test 23 | 9.63 × 10^4^ | 3.61 × 10^3^ | + | + |
| Test 24 | 6.49 × 10^2^ | 6.49 × 10^1^ | + | + |
| Test 25 | 7.04 × 10^4^ | 5.29 × 10^3^ | + | + |
| Test 26 | 5.93 × 10^4^ | 1.48 × 10^3^ | + | + |
| Test 27 | 6.52 × 10^1^ | **—** | + | + |
| Test 28 | 8.57 × 10^3^ | 6.95 × 10^4^ | + | + |
| Test 29 | 1.94 × 10^2^ | 7.53 × 10^1^ | + | + |
| Test 30 | 4.96 × 10^5^ | 3.92 × 10^6^ | + | + |
| Test 31 | 1.04 × 10^5^ | 9.42 × 10^5^ | + | + |
| Test 32 | 3.64 × 10^4^ | 1.42 × 10^5^ | + | + |
| Test 33 | 7.43 × 10^5^ | 8.37 × 10^4^ | + | + |
| Test 34 | 9.68 × 10^3^ | 4.26 × 10^2^ | + | + |
| Test 35 | 1.36 × 10^3^ | 7.53 × 10^4^ | + | + |
| Test 36 | 6.42 × 10^2^ | 8.61 × 10^1^ | + | + |
| Test 37 | 4.82 × 10^3^ | 2.85 × 10^4^ | + | + |
| Test 38 | 1.21 × 10^4^ | 7.32 × 10^4^ | + | + |
| Test 39 | 1.04 × 10^2^ | 6.92 × 10^1^ | + | + |
| Test 40 | 4.51 × 10^2^ | 1.02 × 10^2^ | + | + |
| Test 41-56 | **—** | **—** | **—** | **—** |

Notice: ^a^HBV-RNA-qPCR, the HBV-RNA-qPCR diagnosis was performed using commercially available real-time TaqMan PCR Kit for HBV RNA (SanSure Biotech; Changsha, China). The concentrations of HBV-RNA >50 copies/mL were regarded as positive based on the manufacturer’s recommendations; ^b^HBV-DNA-qPCR, the HBV-DNA-qPCR diagnosis was performed using commercially available real-time TaqMan PCR Kit for HBV DNA (SanSure Biotech; Changsha, China). The concentrations of HBV-DNA >5 IU (~30 copies/mL) were regarded as positive based on the manufacturer’s recommendations.

+, Positive; —, Negative.

**Figure legends**


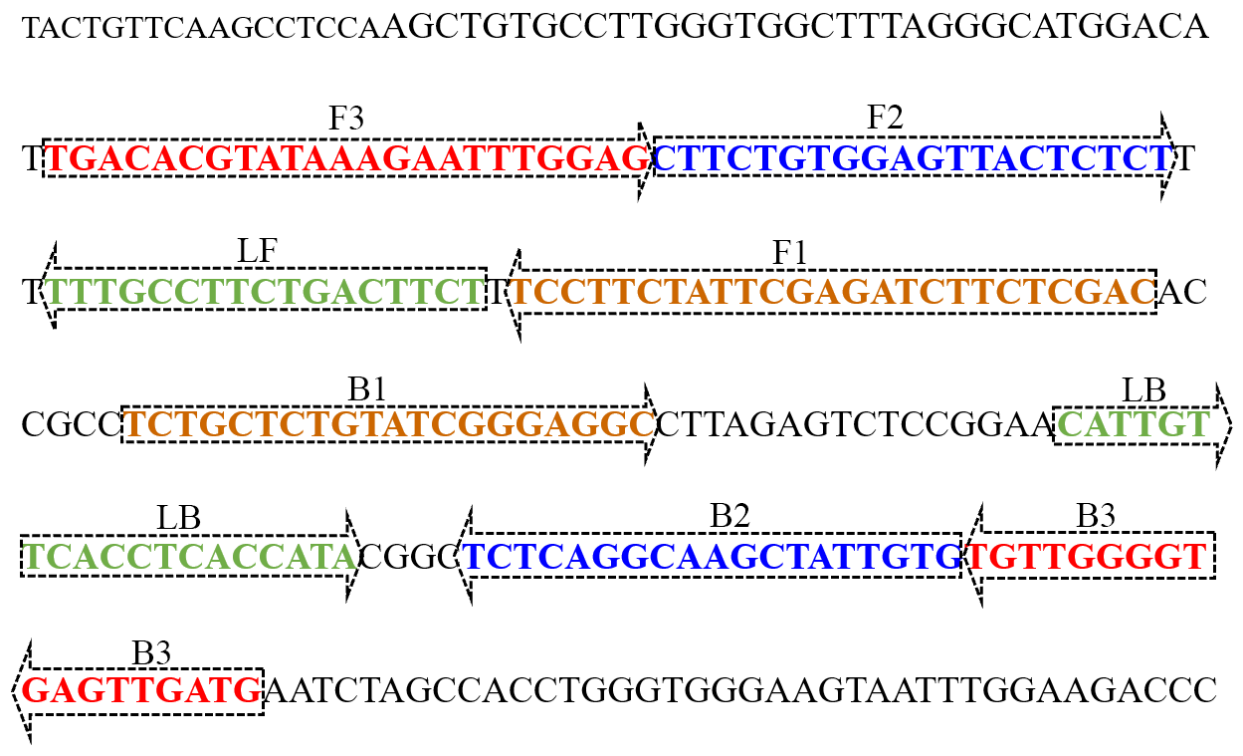


**Fig. S1 Sequences and location of HBV pgRNA gene used to design HBV-RT-LAMP primers**.

The sites of LAMP primers were in boxed. Right arrows and left arrows indicated the sense and complementary sequences which were used in this study, respectively.


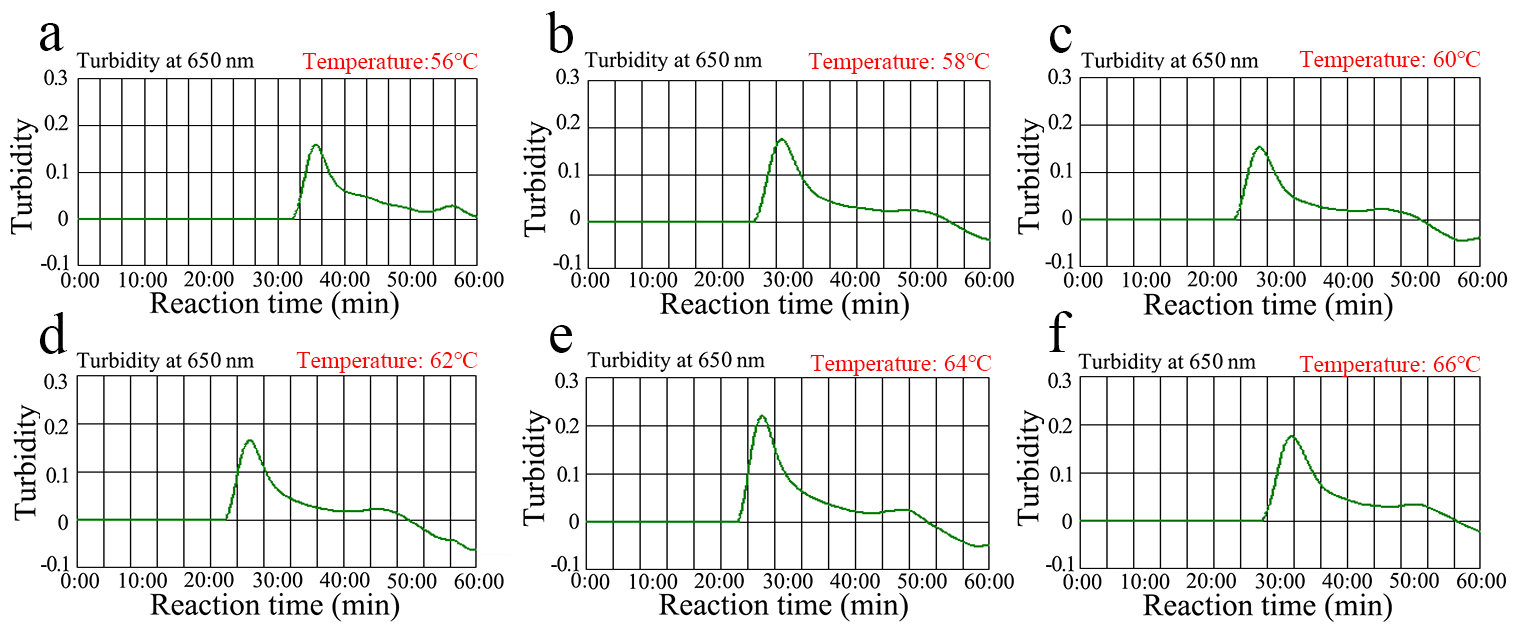


**Fig. S2 Optimizing of the temperature for the HBV-RT-LAMP amplification**

The LAMP amplifications for detection of HBV pgRNA were monitored using real-time turbidity, and the corresponding curves of amplicons were displayed in the graphs. Turbidity > 0.1 indicated a positive value. Six kinetic graphs were obtained at different temperatures (56-66°C, 2°C intervals) with 1×10^4^ copies/mL HBV-RNA standard substance (**a-f**). The graph **e** (64°C) showed the robust amplification.


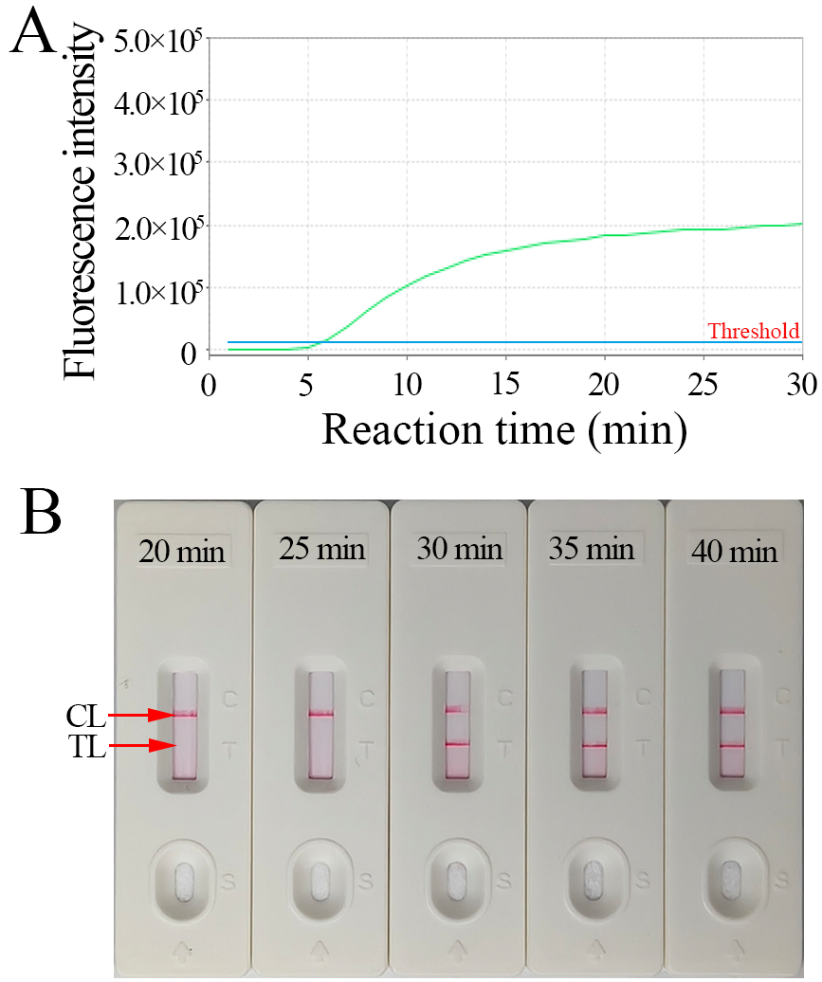


**Fig. S3 Optimizing of the reaction time for HBV-RT-LAMP assay**.

The REF real-time fluorescence (**A**) and AuNPs-LFB (**B**) were used for interpretation of the HBV-RT-LAMP assay. The results showed that the LoD of 50 copies/mL of the HBV-RNA standards were detected when the amplification time was 30 min through visual AuNPs-LFB (**B**), and a stable and robust fluorescent signal (REF) were also appeared within 30 min (**A**). These results indicating that the optimal LoD occurred with a 30 min amplification time.


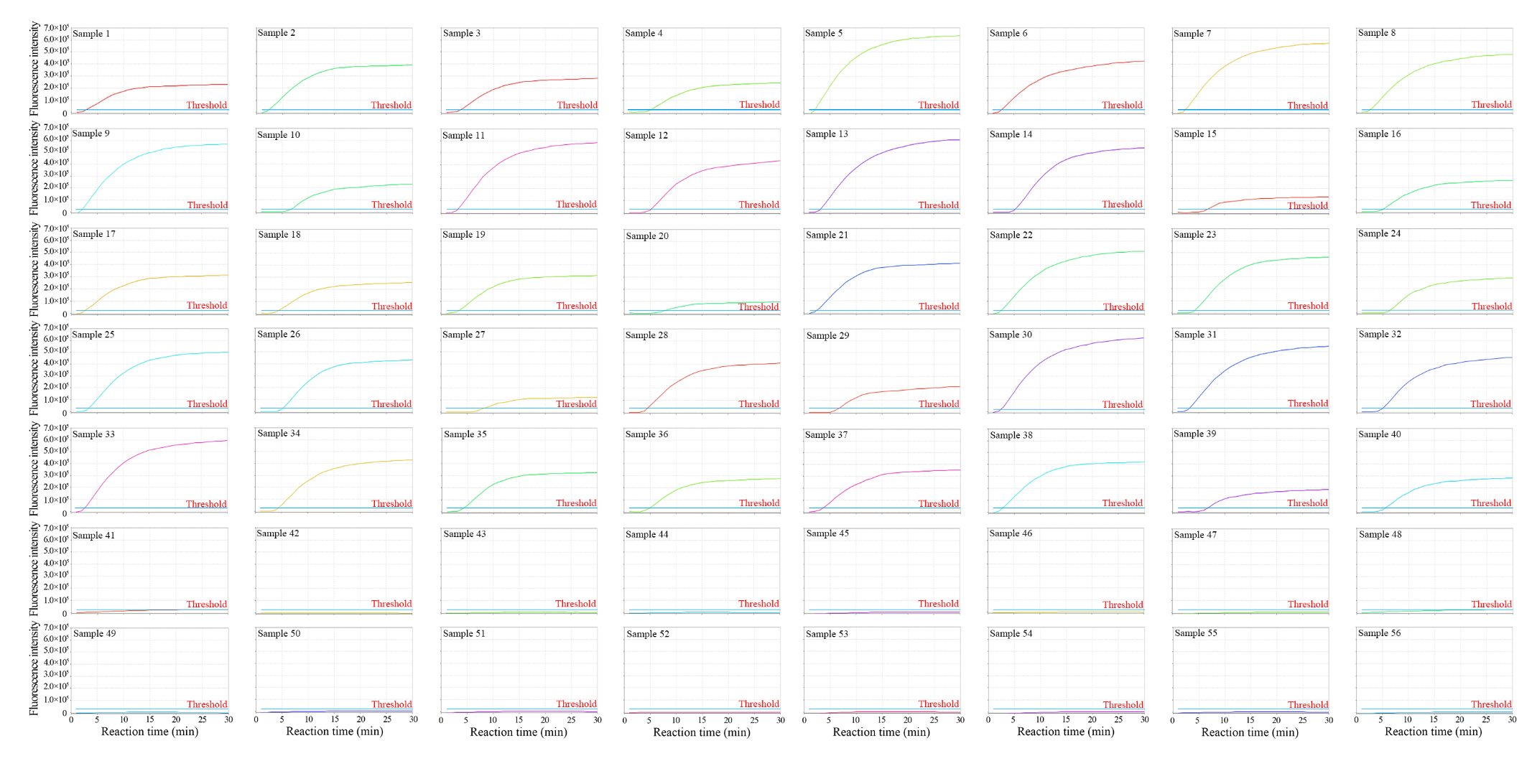


**Fig. S4 The results of HBV-RT-LAMP-REF assay in clinical specimens**.

Forty serum specimens from CHB patients (sample 1 to 40) and sixteen serum specimens from healthy donors (sample 41 to 56) were tested through HBV-RNA-LAMP-REF assay. The results revealed that all forty CHB samples (sample 1 to 40) were diagnosed as positive outcomes, and the other sixteen serum specimens from healthy donors (sample 41 to 56) were tested as negative results.


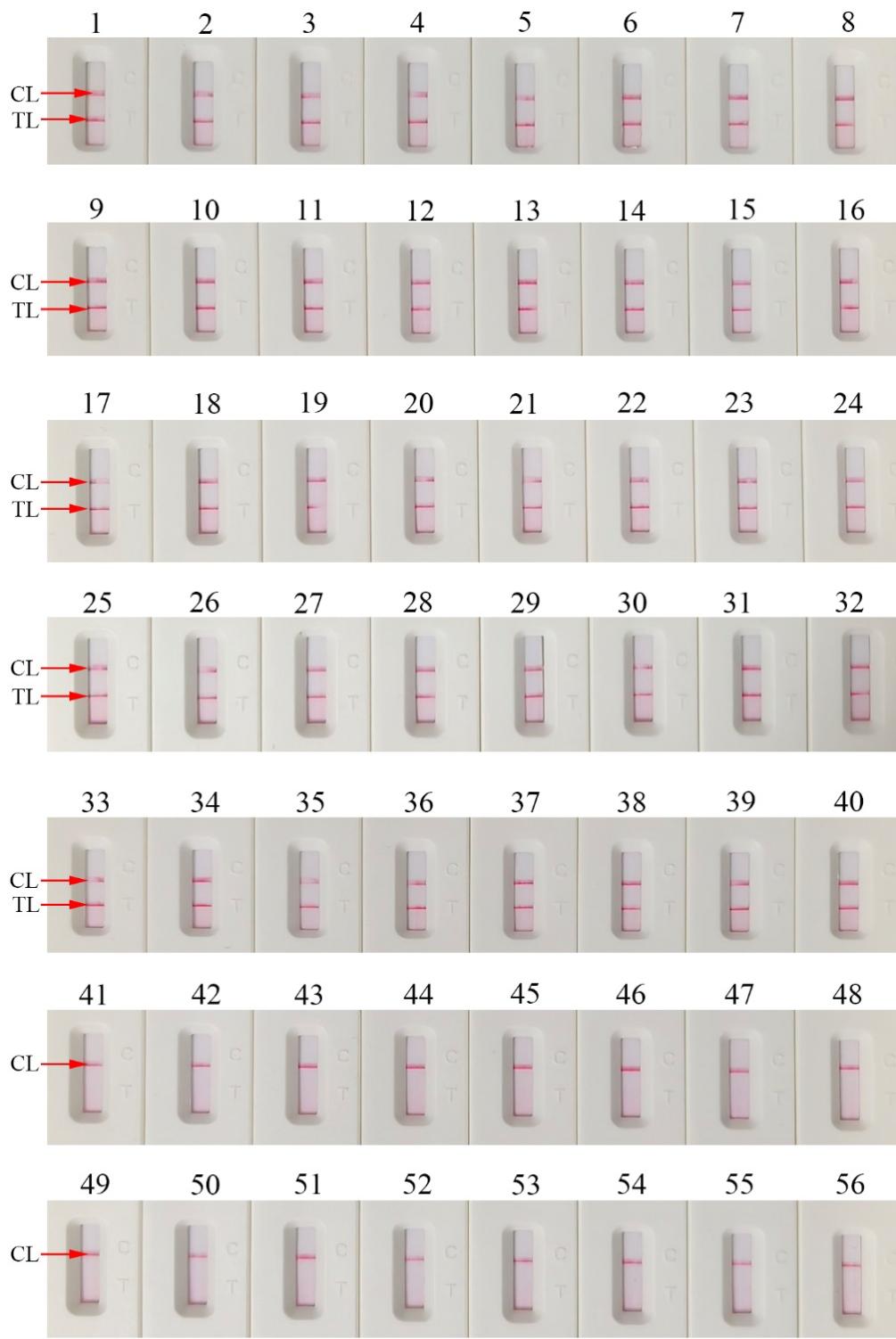


**Fig. S5 The results of HBV-RT-LAMP-AuNPs-LFB assay in clinical specimens.**

Forty serum specimens from CHB patients (sample 1 to 40) and sixteen serum specimens from healthy donors (sample 41 to 56) were tested through HBV-RNA-LAMP-AuNPs-LFB assay. The results showed that all forty CHB samples (sample 1 to 40) were diagnosed as positive outcomes, and the other sixteen serum specimens from healthy donors (sample 41 to 56) were tested as negative results.
